# The Imperial Board of Health of Germany*Besuch des Kaiserlichen Gesundheitsamtes in Berlin. Oesterreichische Vierteljahresschrift für Veterinärkunde. Vol. 60, Part II.

**Published:** 1884-07

**Authors:** 


### Art. XX.—THE IMPERIAL BOARD OF HEALTH OF GERMANY.*

As already indicated by our editorials, this Journal is most determinedly devoted to the advocation of the most exact scientific methods in the study of medicine. We believe that the health of a people is a matter that bears no secondary relation to their monetary prosperity. A nation that is weak physically, or overswept by infectious diseases at intervals, cannot be called prosperous in any sense of the word. The

* Besuch des Kaiserlichen Gesundheitsamtes in Berlin. Oesterreichische Vierteljahresschrift für Veterinärkunde. Vol. 60, Part II.

same is true in a different sense of the animal wealth of a nation. Pests that carry them off in numbers, or even such as appear sporadically in different sections of a country, serve to disturb public confidence in the ability of the government; also, to cause serious disturbances of both our domestic and foreign commerce.

Such diseases of man and animals as have been spoken of cannot be met without an earnest study of every condition under which they appear and extend.

We have made sporadic attempts at such investigations, but they are no more what should be done than if we had made none at all. The people of the United States need just such an institution as the great hygienic system of Germany, with its official representatives in every village, town and city, and also what is fully as important, and for the world more so, than anything else, its investigation station. Hence we can but think that a short description of such an institution will not be without value and interest to our readers.

The first steps towards an Imperial Board of Health for Germany were taken in 1871. Its work was depicted to be "to support the work of medical and veterinary hygienic organization of the land, to gather statistics, and study into the nature and cause of disease." It began its work in 1876. Its officers then were a director, a statistician, and three secretaries. In 1878 a building suitable for the work of the institution was procured and rebuilt. Numerous extraordinary members were added, so that the institution had its special representative in nearly all important places in the Empire.

The *personnel* proper now consists of some twenty members that have their offices in the building at Berlin. There are the director, four regular members, two medical assistants, four bureau officials, three secretaries, three special servants and a porter. The extraordinary members number twenty-three.

The annual funds valued by the government is 124,950 marks, $31,237.50.

The director is the Imperial Privy Councilor, Dr. Struch. The regular working members are Dr. Koch, Dr. Roloff, director of the Veterinary Institute, Dr. Sill, and Prof. Wolfhügel. The number of assistant workers is irregular, according to the demands of the work being carried on; they are selected from among the young and most talented military surgeons.

The institution publishes a weekly report of its doings, giving the statistics of diseases and deaths, especial attention being given to infectious and contagious diseases, as well as any change in the laws with reference to the same, or new instructions with reference to their execution.

It has also published a large volume containing the principal work of the institution up to 1881. Among the original investigations that have thus far been undertaken may be mentioned:

Koch.—Investigations into the nature of pathogenic organisms.

Koch.—Cause of anthrax.

Gaffky.—Experimental septicæmia with reference to its progressive virulence and the development of its germs.

Löffler.—The question of immunity.

Wolfhügel.—On disinfectants and disinfection.

Koch.—On the same.

Proskaner.—On the air with reference to the sulphurous acid contained in it.

Koch and Wolfhügel.—Disinfection with hot air.

Koch, Gaffky and Löffler.—Disinfection with hot water.

Hüppe.—Department of unformed ferments towards high temperatures.

Wolfhügel and Knome.—Action of carbolic acid, etc.

Sell.—Water analyses from a hygienic point of view.

Preusse.—Investigations with milk with reference to the market control.

Wolfhügel and Hüppe.—The penetration of heat into meat while cooking.

Schütz and Löffler.—Experiments with the germ of glanders.

These quotations but indicate the work of an institution which is fast accumulating obligations of the world to its workers and the government that supports them, that no monetary contributions can ever balance. Probably the most noted work emanating from this centre of science has been the discovery of the bacillus, which appears in connection with tuberculosis, and which it would seem is identical in form and characteristics in both human and bovine tuberculosis. In the earlier days of mycological research the nutriment, or medium, that was used by the cultivators of germs were mostly of a fluid nature, but to Koch we owe the establishment of the fact that they can be much more easily and surely developed upon or in substances of a more solid character, which more easily prevents the pollution of such experiments with adventitious elements.

Potatoes have been found to serve the ends desired to the best advantage. Before they can be used they must be thoroughly cleaned and disinfected. To this end they are first placed in a weak solution of corrosive sublimate for a half hour, then in a leaden vessel, the bottom of which is full of apertures, when they are subjected to sterilization in a steaming apparatus and a temperature of 100° C. for several hours. If dry heat is used it has been found that a temperature of 150° C. is necessary to completely sterilize the objects, as moist heat more easily penetrates the substance of solid media than dry.

After these processes are completed the potatoes are segmented with a knife the blade of which has been previously subjected to a red heat. The pieces are then immediately placed under appropriate glass receivers. The germs to be cultivated are then carefully introduced on a platinum needle that has been previously subjected to the action of heat. In conveyance the germs are planted upon distinct parts of the media from which their development takes place. Should the object have become polluted by any adventitious germs, the variations in color and the manner of their development soon tell the story to the eye of the observer.

The bacillus of greenish pus, the B. prodigiosus, which is distinguished by its red color, the potato bacillus, which transforms starch into sugar, the bacillus of anthrax, and various mould fungi, all develop well upon potatoes as a nutrient medium. Paste made from bread is another fine element used for the development of germs. It is prepared by crumbling the bread as finely as possible and then mixing it with water. Otherwise it is treated in the same manner as the potatoes. It is especially adapted to the development of the various varieties of mould fungi, such as aspergillus flavescens, a. fumigatus, a. niger, the penicilli and mucorini. Some of these varieties are of pathogenic importance. They can only be cultivated upon an acid basis which the paste supplies, although by certain morphological or biological metamorphoses their products finally develop upon neutral or alcoholic bases. Gelatin acts as a good nutrient medium for the study of germs that come suspended in the air, the water, or are found in the earth. It can be made by taking one kilogramme of hashed beef and covering it with two litres of distilled water; let it stand for twenty-four hours; to this then add one per cent. pepton, half per cent. salt, and two and a half to three per cent. gelatin. This mixture is to be neutralized with carbonate of soda, and sterilized by heat, as before mentioned; an exposure of two or three hours to a temperature of 150° C. being necessary. In consequence of the coagulation of the albuminous substances in the mixture, it must be filtered in a hot tunnel, that is, a double glass tunnel filled with hot water, the same being kept at a regular temperature by means of a lamp or gas; by this means the development of germs during the process of filtration is prevented. The material left is a clear fluid of yellowish color.

The building devoted to use of the Imperial Board of Health immediately joins the grounds of the Veterinary Institute. In it reside the director, the porter, and other servants. It is supplied with laboratories, rooms for animals for experiments, an autopsy room, a cremation room for the cadavers of animals operated upon, rooms for the various officers, a library, a museum,.a chemical laboratory, a work room, a laboratory for the director, a room for photography, a room for the study of disinfectants, and in fact everything necessary for the development of the important work undertaken by this invaluable institution.

In appropriate relation to the preceding remarks are those of Prof. Frankel on the staining of Koch's bacillus and its semiotic importance in connection with the diseases of the respiratory organs.*

These bacilli have been found to possess certain characteristic reactions towards various staining materials that are valuable aids in their diagnosis and that of certain pulmonary diseases of men and animals. We cannot assert that it is still difficult to stain these bacilli.

---

*Berliner Klinische Wochen Schrift, No. 13, 1884.

They become stained:

1st.—Only with certain aniline colors.

2d.—By ordinary chamber temperature only after the elapse of some time.

3d.—Certain addition to the staining fluids render their action more sure.

Of all the aniline colors, methyl violet and fuchsin offer the most satisfactory results. Both are simple combinations of muriatic acid; both are soluble in water and alcohol, and both color animal tissues, wool and silk, easier than those of vegetable substances—cotton. I have tested over twenty aniline colors in relation to their staining powers upon Koch's bacilli, but none of them bears any comparison to the above in regard to the rapidity and correctness with which they stain the bacilli in question.

The bacilli are the last to become stained among the elements of which a specimen is composed. In ordinary temperature they begin to stain after the elapse of some ten minutes. For diagnostic purposes it is, therefore, better to gently cover such objects.

Koch has mentioned that the staining occurs better and more rapidly in the warm chamber. Rindfleisch warms the object gently over a flame, in order to obtain almost momentary tinction. All authors appear to be united upon this point. They, however, differ considerably as to the adjuvantia to be connected with the staining material. Water colored with alcoholic solution of fuchsin and methyl violet finally color these bacilli as well as concentrated alcohol solutions without the addition of water. Such staining, even with exposure to heat, is not so intensive or general as when certain additions have been made to such solutions. Koch adds a few drops of a weak alcoholic solution; Ehlict recommends the addition of aniline water; Ziel a proportion of a five per cent. solution of carbolic acid; Prior, turpentine; Weigert, ammonia, one-half per cent. solution. In this regard I have made numerous comparative experiments and am convinced that for diagnostic purposes the addition of aniline waters offers the most satisfactory advantages, as such a combination colors the bacilli the most easily and intensively. (Ortho.) Toluidin, which occurs as a polluting element in commercial anilines, as well as by itself, seems to exert an equally favorable influence with aniline. I may remark that I have not been able to gain such satisfactory results from the use of acidulated solutions, with the exception of carbolic acid, as others have reported. When we add an acid to alcalic aniline water, the aniline remains such in the mixture, which cannot be considered acid, notwithstanding it has an acid relation. Fuchsin solutions, treated with acetic acid without the combination of aniline, do not stain the bacilli. They give a black color when exposed to direct light, but with reflected they occasionally have a reddish tinge. Aniline and toluidin do not readily dissolve in water, but very well in alcohol. Aniline water is prepared by mixing thoroughly 5 cms. of aniline oil with distilled water and filtering carefully through a moistened filter. One hundred parts of water by a temperature of 16° C., dissolves 3.11 parts aniline; by 56°, 3.58 parts; by 82°, about 5.18 parts. Aniline water changes with time, but the addition of 5 to 10 per cent. alcohol makes a durable preparation. When one does not desire to filtrate the preparation, we can prepare such a solution by placing 3 cms. aniline oil in 7 cms. alcohol, to which we add 90 cms. water. I can find no reference to the solubility of toluidin in water. So far as I know, it is only one-half as soluble in water as aniline, say 1.5 per cent. This, however, gives a satisfactory solution for staining these bacilli. Concentrated alcoholic solutions of aniline or toluidin do not give satisfactory results. The formula for toluidin should read thus:

R.

| | |
|---|---|
| Toluidin, - - - - | 1.5 |
| Alcohol, - - - - | 8.5 |
| Aq. dist. - - - - | 90. |

Filter.

In either aniline or toluidin water, the aniline colors are, therefore, to be dissolved. The best way to prepare tincture to keep on hand, saturated alcoholic solutions of methyl violet and fuchsin, which are prepared by adding either of these substances to alcohol until a precipitate accumulates in the bottle. This mixture is to be added to the aniline water in a proportion of eleven parts of the former to one hundred of the latter. It is to be recommended that each solution be kept in bottles with dropper stopples, and the staining mixture prepared in small quantities only when wanted for immediate use.

When I wish to prepare such a mixture I place 5 cms. of the aniline or toluidin in water in a test tube and warm it, and add it to the correct proportion of the alcoholic preparations, which have been previously placed in a watch glass; in this mixture I place the covering glass, having the object to be stained on it, the bacilli becoming stained in two minutes, though we may leave them in the staining fluid five to ten if we desire. When we have thus stained our bacilli our work is but half done; it becomes necessary for us to differentiate them. They undoubtedly stain less easily than others, but when they do the coloring is remarkably pure and well defined. When once stained they retain their color much more intensively than any other elements in the specimen. Agents which cause the decoloration of other elements are energetically resisted by the bacilli. Among such may be mentioned acids, alcohol, other aniline colors, etc. When nitric acid is used for a test, the acid combinations of fuchsin and methyl violet become a gold and green color, respectively. This phenomenon has caused much discussion. The Koch bacilli finally he recolored in diluted nitric acid, 1:3—; this takes place easier than in glacial acid. With the exception of Rindfleisch all authors agree in treating such preparations a second time with some tinction having the strongest possible contrast with that first used, for instance: bacilli that have been first treated with mythyl violet, at the second staining with brown—vesuvin; fuchsin bacilli with some blue tinction—methylen-blue; or green, malachite green—aethyl green. This contrast tinction is of great value in the diagnosis, as it sometimes happens that some accidentally present bacilli are not decolored by the acid, but the second staining relieves all embarrassment.

The various other tinctions may be prepared thus:

*Blue:* 50 per cent. alcohol, 30 aqua, 20 nitric acid, with as much methyl blue as will dissolve after repeated shaking.

*Brown:* 70 per cent. alcohol, 30 nitric acid, as much vesuvin as will dissolve.

*Green.*—50 per cent. alcohol, 20 aqua, 30 glacial acid and as much malachite or aniline green as dissolves. Filter all of them.

If we place the discolored covering glass in such a preparation, its material becomes again stained with the blue mixture in one minute, with the brown in one and a half to two minutes; 3.5 cm. of such solutions should be used. Following this, the covering glasses should be placed in water of weak acid solution (1 per cent. acetic acid), and washed in a 50 per cent. alcoholic solution, and well dried. In this way we can prepare a double colored object in about four minutes.

When we ask what semiotic importance has the examination of sputa for bacilli, we must answer that where we find them in sputum, a destructive process, due to their influence, is present in the respiratory tract.

We should not say that tubercular, but bacilli, destruction is occurring; hence the name phthisis is far preferable for such processes than tuberculosis.

The positive discovery of bacilli in such sputum assures the diagnosis in a manner not to be attained in any other way, as is illustrated in the discovery of such bacilli in the differentiation between pulmonary syphilis and phthisis; or in the difference between the bacilli of fibrinous pneumonia and phthisis, as is discoverable by the tinction methods already given. Continued examination of the sputum should be most exactly made before a diagnosis is determined upon in any case of doubt. Gaffky examined 982 examples of sputa from the same number of patients, and found Koch's bacilli in 938 cases, with a negative result of only 4.5 per cent. It is only in doubtful cases that more than three examinations of the sputum is necessary.

When we have taken all necessary precautions, we are justified in assuming that no destructive processes, due to bacilli, are present when negative results follow our examinations.

